# A Novel Role for MAPKAPK2 in Morphogenesis during Zebrafish Development

**DOI:** 10.1371/journal.pgen.1000413

**Published:** 2009-03-13

**Authors:** Beth A. Holloway, Sol Gomez de la Torre Canny, Ying Ye, Diane C. Slusarski, Christina M. Freisinger, Roland Dosch, Margaret M. Chou, Daniel S. Wagner, Mary C. Mullins

**Affiliations:** 1 Department of Cell and Developmental Biology, University of Pennsylvania School of Medicine, Philadelphia, Pennsylvania, United States of America; 2 Department of Biochemistry and Cell Biology, Rice University, Houston, Texas, United States of America; 3 Department of Biology, The University of Iowa, Iowa City, Iowa, United States of America; Stanford University School of Medicine, United States of America

## Abstract

One of the earliest morphogenetic processes in the development of many animals is epiboly. In the zebrafish, epiboly ensues when the animally localized blastoderm cells spread, thin over, and enclose the vegetally localized yolk. Only a few factors are known to function in this fundamental process. We identified a maternal-effect mutant, *betty boop* (*bbp*), which displays a novel defect in epiboly, wherein the blastoderm margin constricts dramatically, precisely when half of the yolk cell is covered by the blastoderm, causing the yolk cell to burst. Whole-blastoderm transplants and mRNA microinjection rescue demonstrate that Bbp functions in the yolk cell to regulate epiboly. We positionally cloned the maternal-effect *bbp* mutant gene and identified it as the zebrafish homolog of the serine-threonine kinase Mitogen Activated Protein Kinase Activated Protein Kinase 2, or MAPKAPK2, which was not previously known to function in embryonic development. We show that the regulation of MAPKAPK2 is conserved and p38 MAP kinase functions upstream of MAPKAPK2 in regulating epiboly in the zebrafish embryo. Dramatic alterations in calcium dynamics, together with the massive marginal constrictive force observed in *bbp* mutants, indicate precocious constriction of an F-actin network within the yolk cell, which first forms at 50% epiboly and regulates epiboly progression. We show that MAPKAPK2 activity and its regulator p38 MAPK function in the yolk cell to regulate the process of epiboly, identifying a new pathway regulating this cell movement process. We postulate that a p38 MAPKAPK2 kinase cascade modulates the activity of F-actin at the yolk cell margin circumference allowing the gradual closure of the blastopore as epiboly progresses.

## Author Summary

One of the earliest cell movement processes in the development of many animals is epiboly. In the zebrafish, epiboly ensues when the blastoderm cells spread over and enclose the yolk cell. Only a few factors are known to function in this fundamental process. We identified a maternal-effect mutant, *betty boop* (*bbp*), which displays a novel defect in epiboly, wherein the blastoderm margin constricts dramatically, precisely when half of the yolk cell is covered by the blastoderm, causing the yolk cell to burst. We demonstrate that Bbp functions in the yolk cell to regulate epiboly. We positionally cloned the *bbp* mutant gene and identified it as the serine-threonine kinase Mitogen Activated Protein Kinase Activated Protein Kinase 2, or MAPKAPK2, which was not previously known to function in embryonic development. We show that the regulation of MAPKAPK2 is conserved within a p38 MAP kinase pathway, thus identifying a new pathway in the regulation of this fundamental cell movement process. We postulate that a p38 MAPKAPK2 kinase cascade modulates F-actin contraction at the yolk cell margin circumference, allowing the gradual closure of the cells over the yolk cell as epiboly progresses.

## Introduction

**Figure 1:**
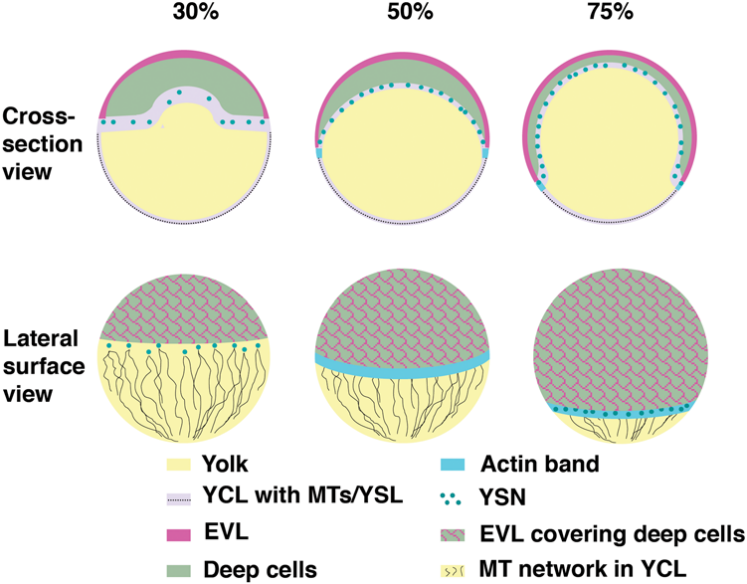
Schematic of epiboly progression. Epiboly begins when the yolk (yellow) domes toward the animal pole (top), concomitant with radial intercalation of the deep cells of the blastoderm (green) to cover 30% of the embryo surface. At 30% epiboly, the yolk syncytial nuclei (YSN, dark-green circles) are maintained within the yolk syncytial layer (YSL) beneath the blastoderm and are associated with microtubules (black lines, surface view) that are oriented toward the vegetal pole within the very thin cortical, yolk cytoplasmic layer (YCL, light blue in cross-section). The enveloping layer (EVL, pink) forms a thin epithelial-like sheath on the surface of the deep cells and is connected to the YSL at the cell margin. The YCL cannot be distinguished from the yolk when viewed from the lateral surface. At 50% epiboly, the EVL and deep cells cover 50% of the embryo and lie more vegetally than the YSN, masking the YSN in the lateral surface view. An actin band (turquoise) forms in the YSL just vegetal to the margin at 50% epiboly and likely functions as a contractile mechanism to close the blastopore throughout the remainder of the epiboly process. By 75% epiboly, the YSN are located beneath the blastoderm, as well as vegetal to it (surface view). The blastopore is the exposed vegetal yolk, which continually decreases in circumference as epiboly progresses.

Early embryonic development is marked by cellular movements that ultimately generate the shape of the embryo in a process known as morphogenesis. One of the earliest morphogenetic events in many animals is the process of epiboly, whereby embryonic tissues spread and thin [1]–[5]. In the zebrafish embryo, three distinct cell layers lying at the animal pole of the embryo undergo epiboly: the enveloping layer (EVL) and yolk syncytial layer (YSL), both of which are extraembryonic, and an intermediate deep cell layer that forms the embryo proper (Fig. 1). About 1 hour after the mid-blastula transition, the morphogenetic process of epiboly begins. The deep blastomeres radially intercalate, while the underlying yolk moves animalward in a process called doming. At completion of this initial phase of epiboly, an inverted bowl-shaped blastoderm covers ∼50% of the yolk surface, referred to as the 50% epiboly stage (Fig. 1). During the second phase of epiboly, the deep cells begin gastrulation cell movements converging dorsally and undergoing involution/ingression movements to form the germ layers [5]. At the same time, epiboly continues with all three cell layers spreading over the yolk to the vegetal pole of the embryo, ultimately resulting in the complete internalization of the yolk [6]. The morphogenetic process of epiboly also occurs in numerous other vertebrates and invertebrates, including amphibia, sea urchins, and C.elegans [1]–[4].

The YSL actively participates in epiboly. Within the YSL, microtubule organizing centers associated with the yolk syncytial nuclei (YSN) extend microtubule arrays vegetally into the cortical yolk cytoplasmic layer (YCL) (Fig. 1). Ablation of microtubules with UV treatment or nocodazole slows or arrests epiboly progression [7],[8]. Studies of YSN movements suggest that movement of blastomeres and YSN are coordinated [9]. Although the mechanism remains unknown, E-cadherin is required for the coupling of the deep cells to the YSL and EVL in coordinating this movement between tissue layers in zebrafish [10]–[12]. In Xenopus fibronectin-integrin cell adhesion interactions act in radial intercalation during epiboly [13]. As the EVL and blastoderm cells move over the yolk, the yolk cell membrane is actively removed via endocytosis [14]. Also within the YSL is an actin band, first identified in *Fundulus heteroclitus*, which is required for epiboly movements and is postulated to close the blastopore (the uncovered vegetal yolk surface) as epiboly progresses (Fig. 1) [15]–[17].

Although epiboly is a fundamental morphogenetic process, only a handful of factors have been identified regulating this process. To identify additional molecular regulators, we investigated the zebrafish maternal-effect mutant, *betty boop (bbp)*, which displays a novel defect in epiboly [18],[19]. In *bbp* mutants the leading edge of the blastoderm constricts dramatically at 50% epiboly causing the yolk cell to burst. This defect in epiboly is not seen in other mutants or by pharmacological treatments, suggesting that a novel aspect of epiboly is affected. Through whole blastoderm transplants and mRNA microinjection rescue, we determined that Bbp functions in the yolk cell. Consistent with it having a novel function in epiboly, we found that microtubules, actin band formation and endocytosis in the YSL appear normal prior to the onset of the phenotype. We positionally cloned the *bbp* mutant gene and identified it as the zebrafish homolog of Mitogen Activated Protein Kinase Activated Protein Kinase 2 (MAPKAPK2). Mutation of the p38 MAP kinase phosphorylation sites in MAPKAPK2 implicates p38 in regulating MAPKAPK2 function. Expression of a dominant-negative p38 MAP kinase demonstrates that it functions in a similar manner to MAPKAPK2 in epiboly. Furthermore, we found a dramatic increase in calcium release in *bbp* mutants, possibly reflecting altered actin contraction. Thus, we identified the p38 MAPAPK2 pathway as a new regulator of the fundamental morphogenetic process of epiboly. We propose that the p38 MAPKAPK2 kinase cascade modulates actin contraction at the blastoderm margin to close the blastopore during normal epiboly progression.

## Results

### *bbp* Mutants Display Precocious Blastopore Constriction

**Figure 2:**
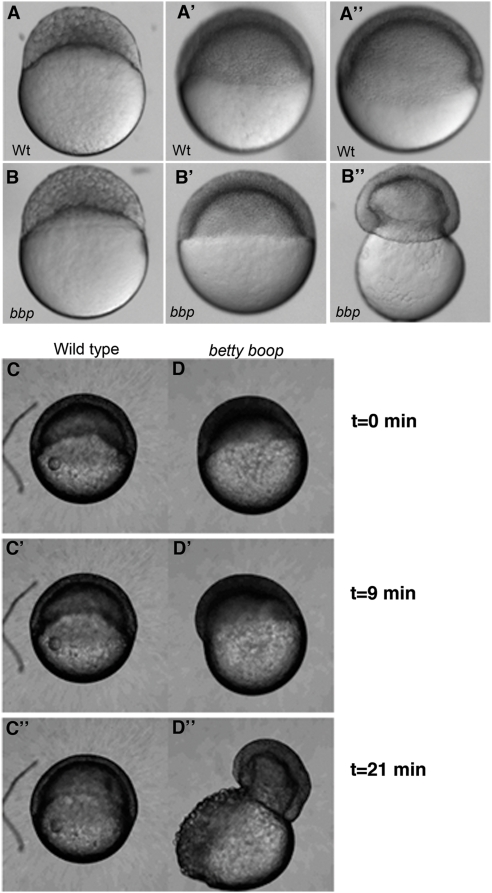
*bbp* embryos constrict at the margin at 50% epiboly, bursting the yolk. Still images of WT and *bbp* embryos at the 1000-cell stage (A, B), 50% epiboly (A′, B′), and 50% epiboly at the burst (A″, B″). *bbp* embryos constrict just as they reach 50% epiboly. (C and D) Selected frames from time-lapse movies of WT and *bbp* embryos showing that the constriction occurs rapidly once the embryos have reached 50% epiboly.

In a maternal-effect screen, we identified a mutant, *bbp*, which displays a striking morphogenesis defect in epiboly [18]. *bbp* mutant embryos appear to develop normally until 50% epiboly (Fig. 2 A,B); however, just as the blastoderm cells reach 50% epiboly, the equator constricts around its circumference, causing the yolk cell to burst (Fig. 2 B″). This defect is a 100% penetrant, maternal-effect phenotype, in which all embryos from homozygous females are mutant (hereafter called *bbp* mutant embryos), irrespective of their paternal genotype. Time-lapse microscopy of mutant (n = 9) and wild-type (WT) (n = 9) embryos (Fig. 2 C–D) shows that *bbp* embryos undergo abnormal shimmying movements periodically during blastula stages. That is, rapid, abnormal movements of large regions of the blastoderm, which increase in amplitude until the constriction and bursting of the yolk (Video S1).

**Figure 3:**
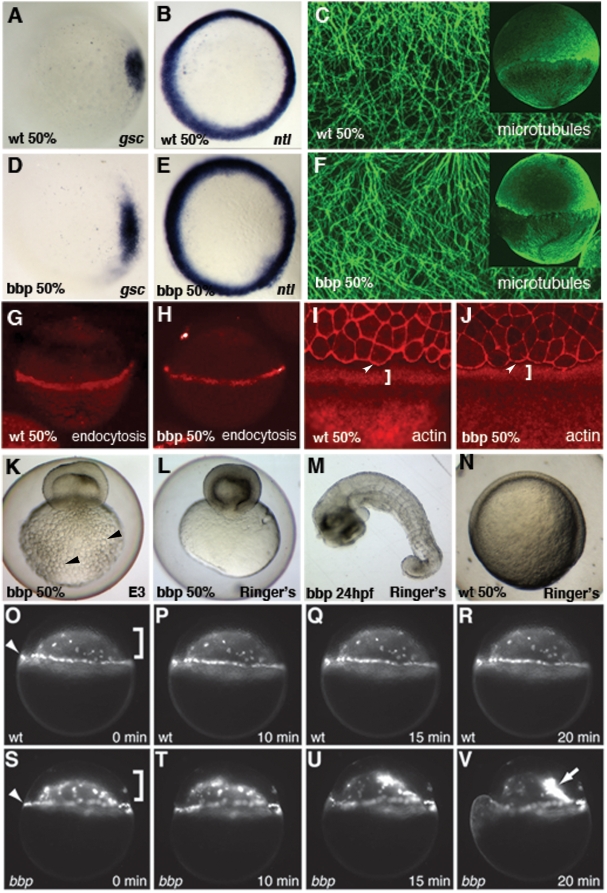
Characterization of *bbp* phenotype. *In situ* hybridization of *goosecoid* (*gsc*) in WT (A) and *bbp* (D) and *no tail* (*ntl*) in WT (B) and *bbp* (E) embryos at 50% epiboly. Microtubule array forms normally in *bbp* embryo (F) at the approach to 50% epiboly as in WT (C). G, H Internalization of Rhodamine-dextran through endocytosis in the YSL at 50% epiboly in WT (G) and a *bbp* (H) embryo. Rhodamine-Phalloidin staining in WT (I) and *bbp* (J) embryos at 50% epiboly. White brackets show punctate actin band in YSL, arrowheads indicate actin at EVL margin. Hypertonic Ringer's solution extended development of constricting *bbp* embryos. *bbp* embryo ni E3 medium constricts at 50% epiboly and yolk globules (black arrowheads) burst through the yolk membrane (K). *bbp* embryo reared in hypertonic medium constricts, while maintaining yolk membrane integrity (L), allowing the blastoderm to heal and survive to 24 hpf (M). Mutants can undergo gastrulation movements and generate a general body plan at 24 hpf. WT sibling reared in hypertonic medium develops normally, shown at 50% epiboly (N). YSN of WT embryo labeled with Sytox Green (O–R); both I-YSN (bracket) and E-YSN (arrowhead) change little during this short time interval. (S–V) *bbp* I-YSN are initially distributed normally under the blastoderm, but retract rapidly to a small region of the I-YSL (arrow) when the margin constricts, just before yolk cell rupture.

We investigated if defects in patterning could account for the *bbp* phenotype. We found that the expression of *goosecoid* (Fig. 3 A, D), a dorsal organizer marker, and *no tail* (Fig. 3 B, E), a mesodermal marker, was normal in *bbp* embryos (n = 10, 13 respectively). Analysis of *bmp4* and *eve1* expression in ventral and ventrolateral regions, as well as *foxb1.2* [20] in dorsal regions confirmed that patterning is normal in *bbp* embryos (data not shown). Thus, the defect in *bbp* appears to be specific to the morphogenetic process of epiboly.

Proper epiboly progression depends on microtubules that are present in the yolk cell [7],[8]. We analyzed microtubule array formation in the YCL (yolk cytoplasmic layer) at 50% epiboly just prior to bursting. Anti-tubulin stainings showed that microtubules are properly formed and robust in mutant embryos (Fig. 3 F, n = 23), similar to WT embryos (Fig. 3 C, n = 17). Thus, microtubule array formation does not appear defective in *bbp* embryos.

As epiboly progresses, the yolk cell membrane adjacent to the advancing blastoderm cells is removed via endocytosis. This process decreases the yolk cell membrane during epiboly, as the deep cells, EVL and YSN move over the yolk cell, and may drive their vegetal movement [14],[16]. We found that *bbp* mutant (n = 8) and WT (n = 10) embryos both internalized Rhodamine-dextran dye (Fig. 3 G,H), indicating that endocytosis was normal and is unlikely the cause of the epiboly defect.

It was unclear if the primary cause of the bursting of *bbp* embryos is the marginal constriction or a loss of yolk membrane integrity, which secondarily leads to the buckling of the blastoderm margin. To distinguish between these possibilities, we incubated WT and *bbp* embryos in hypertonic media to stabilize the yolk membrane in an attempt to prevent it from failing during the manifestation of the phenotype. The hypertonic medium caused no defects in WT embryos (Fig. 3 N) and stabilized the yolk membrane of *bbp* mutants, as evident by the lack of yolk globules protruding through the membrane (Fig. 3 K, L arrowheads). Importantly, despite the stabilized membrane, *bbp* embryos continued to constrict around their circumference at 50% epiboly in the hypertonic medium without causing the yolk to burst (Fig. 3 L, n = 56). Eventually the blastoderm pinched off from the vegetal yolk and healed, and the embryo continued to develop to 1 day post fertilization (dpf) (Fig. 3 M). This result indicates that the bursting phenotype results from a mechanical constriction, rather than from a yolk membrane integrity defect.

It is thought that a slow and controlled constriction of an actin ring present in the YSL closes the blastopore as epiboly progresses in the Fundulus embryo [15]. Similarly, in zebrafish a punctate actin band forms just vegetal to the blastoderm/EVL margin in the yolk cell at the 50% epiboly stage [16]. This actin band coincides with the region of yolk cell membrane endocytosis [16] and the marginal constriction of EVL cells during epiboly [21]. Phalloidin staining shows that an F-actin band forms in the YSL and at the EVL margin of *bbp* embryos (Fig. 3 J, n = 15), as in WT embryos (Fig. 3 I, n = 20). However, it remains unclear if the F-actin functions normally during epiboly.

We investigated the timing of the marginal constriction in *bbp* mutant embryos to distinguish between two alternative defects. One possibility is that the vegetal-ward movement of the blastoderm is arrested in *bbp* embryos, while blastopore closure is unaffected, thus causing the observed marginal constriction as the blastopore gradually tries to close in the absence of vegetal-ward cell movement. Such a constriction defect is seen in a subset of embryos in which microtubule function is blocked [7],[8] and in embryos depleted of Mtx2, a presumptive transcription factor acting in the yolk cell to regulate epiboly [22],[23]. Alternatively, the marginal constriction could arise due to too rapid closing of the blastopore, i.e. precocious blastopore closure, caused by unregulated actin constriction. We found that the margin does not constrict gradually over a ∼3 hour period as in WT, but instead occurs rapidly within a 20 to 30 minute period in *bbp* mutants (Fig. 2 and Video S1). This result is consistent with a model in which the blastopore closes precociously in *bbp* mutants through an unregulated marginal F-actin constriction.

We examined the behavior of the yolk syncytial nuclei (YSN) in *bbp* mutant embryos to determine if YSL morphogenesis is affected. Below the blastoderm at 50% epiboly, the internal YSN (I-YSN) are widely distributed, while at the margin the external YSN (E-YSN) are more densely packed [7]–[9]. In time-lapse analysis of fluorescently labeled nuclei, we observed a normal distribution of the I-YSN during epiboly in *bbp* mutant and WT embryos. However, following a strong shimmying movement and coincident with the onset of the marginal constriction shortly before *bbp* mutants burst, the I-YSN withdrew from the location of a strong shimmying movement and collapsed into a small area of the I-YSL (Fig. 3 O–V, Video S2, S3). The E-YSN remained in place until the yolk cell burst minutes later. Thus, YSN epiboly movements appeared normal in *bbp* mutants until 50% epiboly, when strong shimmying movements and the marginal constriction likely cause them to behave abnormally secondarily.

### *bbp* Mutants Display Aberrant Calcium Dynamics

**Figure 4:**
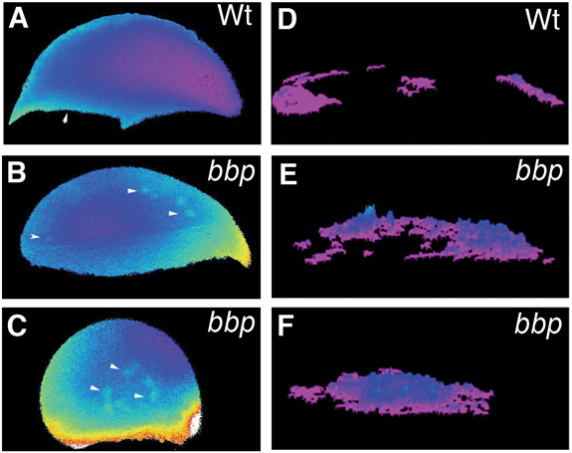
Ectopic calcium release. Selected frames of ratiometric images of WT (A) and *bbp* at early epiboly (B) and *bbp* at 40% epiboly (C). Algorithm of the calcium release representing the frequency and location of fluxes for the duration of the time course of early WT embryo in A (D), early *bbp* embryo in B (E), and 40% epiboly *bbp* embryo in C (F). Composites represent data collected over 1 hour of imaging of an individual embryo.

During early epiboly stages, calcium levels are low throughout the embryo; however, beginning at 50% epiboly calcium levels become dynamic and are required for formation of the yolk cell actin band and epiboly progression [16], [24]–[26]. We investigated if calcium dynamics are altered in *bbp* mutants during epiboly, which could reflect a change in the yolk cell F-actin function. We examined calcium dynamics by ratiometric imaging with the fluorescent calcium indicator, Fura-2. Time-lapse microscopy and transient composite analysis of calcium activity showed a dramatic increase in calcium release in *bbp* mutants (n = 7) compared to WT embryos (Video S4 and S5). During early epiboly when doming of the yolk occurs, calcium activity is maintained at a sustained level in WT embryos (Fig. 4 A, arrowhead), whereas in age-matched *bbp* embryos, ectopic calcium release activity was observed (Fig. 4 B, arrowheads). This ectopic release increased in frequency and intensity as epiboly progressed (Fig. 4 C, arrowheads) until eventual bursting. Analysis of calcium flux throughout early epiboly in a composite pseudo-colored image shows low calcium activity at the margin in WT (Fig. 4 D), which is clearly increased in *bbp* embryos (Fig. 4 E, F). Thus the increased calcium release may lead to increased contraction of F-actin at the yolk margin and the dramatic constriction observed in *bbp* mutant embryos.

### The YSL Is the Critical Domain of Bbp Function

**Figure 5:**
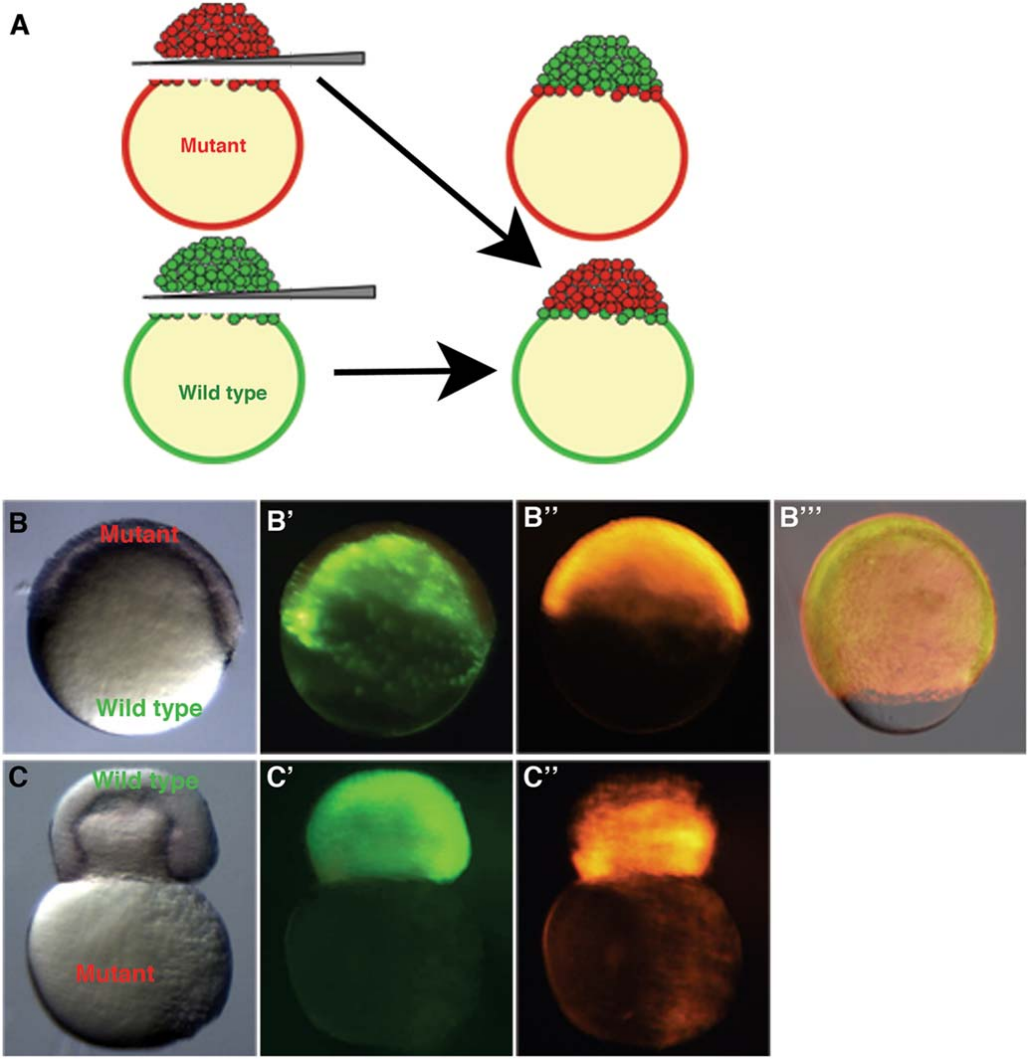
Whole blastoderm transplants show yolk cell domain of function. Embryos were injected with rhodamine-dextran (*bbp*) or sytox green dye (WT) at the 1-cell stage. Blastoderms were separated from yolks and re-adhered creating chimeric embryos. (A) Schematic representation of the blastoderm transplant. A chimeric embryo (B–B′″) containing a WT yolk (B′) and *bbp* blastoderm (B″) progresses through epiboly properly (B′″, 80% epiboly). A chimeric embryo (C–C″) containing a WT blastoderm (C′) and *bbp* yolk (C″) constricts at 50% epiboly, showing that Bbp functions in the yolk cell during epiboly. Bright field images of chimeric embryos, B, C. WT derived tissue, B′, C′. *bbp* derived tissue, B″, C″. Merge of images at 80% epiboly in B′″.

The Bbp protein may function in the yolk cell, deep cells or EVL. To determine the embryonic domain in which Bbp functions, we performed whole blastoderm transplants to separate yolk, YSL, and YCL structures from the deep cells of the blastoderm and EVL [27]. WT blastoderms were then placed on *bbp* yolks, and vice versa (Fig. 5 A). Chimeric embryos containing WT yolk and mutant blastoderm completed epiboly (Fig. 5 B–B′″, n = 5) and were viable through at least 6 dpf (data not shown). In contrast, chimeric embryos containing mutant yolk and WT blastoderm constricted at the equator and burst at 50% epiboly (Fig. 5 C–C″, n = 7), similar to *bbp* embryos. These data indicate that Bbp functions in the yolk cell to regulate epiboly, consistent with it modulating the closing of the blastopore via actin constriction within the yolk cell.

### *bbp* Encodes MAPKAPK2

**Figure 6:**
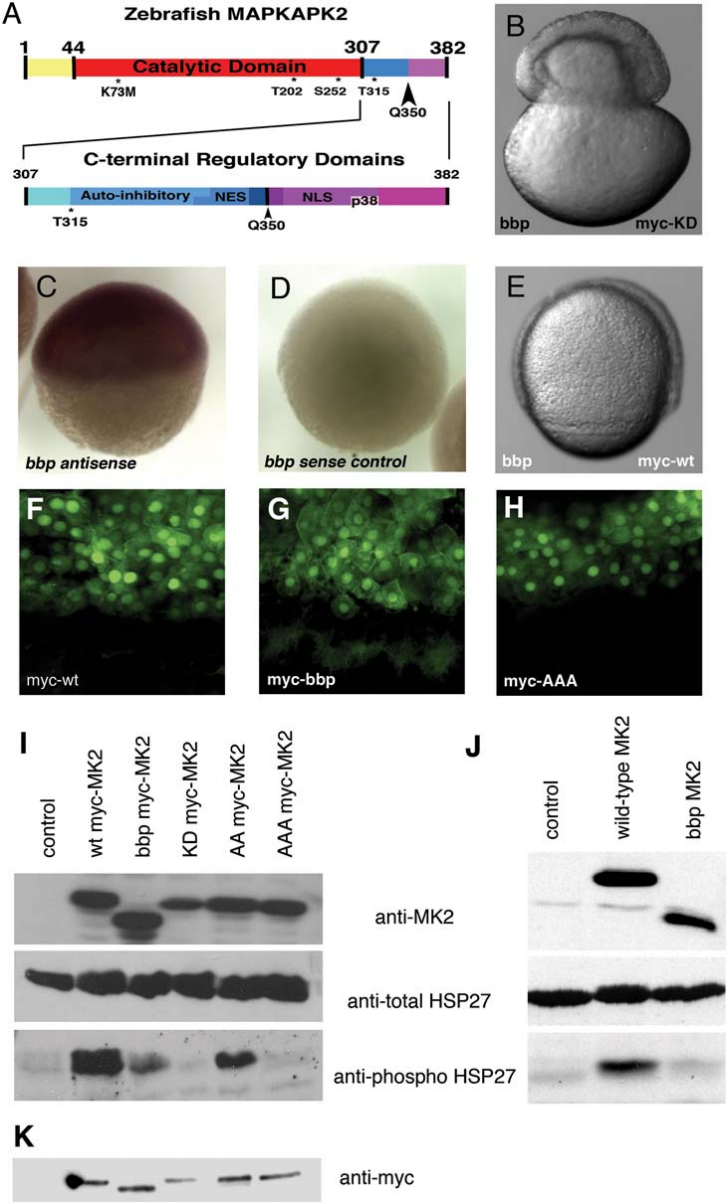
Zebrafish MAPKAPK2 protein schematic, sub-cellular localization, and kinase activity. (A) Top panel: Full length protein of 382 amino acids, with catalytic domain (red) adjacent to the carboxy-terminal regulatory domains (blue, purple). Residues T202, S252, and T315 (*) are phosphorylated by p38 MAPK. K73M is the kinase dead mutation generated. The Q350 residue (black arrowhead) is mutated to a premature stop codon in *bbp*, thus creating a truncated protein lacking the most carboxy-terminal regulatory domains (purple). Bottom panel: Scale representation of carboxy-terminal regulatory domains, including the auto-inhibitory helix that slightly overlaps an NES, and the NLS that also contains residues required for p38 MAPK docking (p38). (B) *bbp* embryo injected with kinase-dead full-length myc-tagged MAPKAPK2 (MK2), which does not rescue; shown at 50% epiboly when the constriction initiated. (C,D) Wholemount in situ hybridization of MK2 (*bbp*) antisense and control sense probes at 50% epiboly in WT embryos. (E) *bbp* embryo injected with full-length myc-tagged MK2 mRNA at the 1-cell stage is rescued; shown at 75% epiboly. (F–H) Anti-myc antibody staining of WT (*bbp*/+) embryos expressing WT (F), *bbp* (G), and a non-phosphorylatable T202A/S252A/T315A triple mutation (H) of myc-tagged MK2 (100 pg mRNA injected). I. Western blot of HeLa cell extracts expressing a vector control (lane 1), full-length myc-tagged MK2 (lane 2), truncated myc-tagged Bbp MK2 (lane 3), full-length kinase dead myc-tagged MK2 (lane 4), T202A/T315A myc-tagged MK2 (lane 5), T202A/S252A/T315A myc-tagged MK2 (lane 6). (J) Western blotting of HeLa cell extracts expressing a vector control (lane 1), full-length untagged MK2 (lane 2), or truncated untagged Bbp MK2 (lane 3). (I, J) HeLa cell extract blots were probed for total MK2 protein (MK2) (top panels), total HSP27 protein (middle panels), and phosphorylated HSP27 protein (bottom panels). (K) Western blot probed with anti-myc of embryo extracts either uninjected (lane 1) or expressing myc-tagged MK2 constructs, as described in I.

To identify the molecular nature of *bbp*, we mapped the *bbp* mutation to the centromere of chromosome 11 using SSLP (simple sequence length polymorphic) markers [28] in bulk segregational analysis of homozygous mutant versus WT sibling adult female fish. The interval was narrowed by fine recombination mapping using more than 1100 meiotic events to a 900 kb interval based on the Sanger Centre zebrafish genome sequence (www.ensembl.org/Danio_rerio). Ten novel ESTs and one known cDNA were present in the critical interval. RT-PCR of ovary cDNA identified 7 ESTs and the one known cDNA as maternally-supplied RNAs (data not shown). Systematic sequencing of these genes from WT and *bbp* mutant ovary cDNA identified a non-sense mutation (Fig. 6A) in the known serine-threonine kinase gene, Mitogen Activated Protein Kinase Activated Protein Kinase 2 (MAPKAPK2). Sequence analysis of the genomic region of the parental mutagenized fish demonstrated that this mutation did not exist in the background and thus was induced by the ENU mutagenesis. The mutation introduces a premature stop codon, predicting a carboxy-terminal 33 amino acid truncation, which removes an identified nuclear localization signal and a p38 docking domain (Fig. 6A) [29],[30]. The MAPKAPK2 transcript is present maternally in the egg and is found ubiquitously in the blastoderm through the 50% epiboly stage (data not shown, Fig. 6C,D).

The *bbp* phenotype could be rescued by injection of 20 pg of WT MAPKAPK2 mRNA at the 1-cell stage (n>500, 100% rescue), demonstrating that MAPKAPK2 is the gene defective in *bbp* mutants. This is among the first maternal-effect mutant genes to be cloned positionally in a vertebrate. Importantly, injection of WT MAPKAPK2 mRNA into the YSL at the 512- to 1000-cell stage rescued the constriction defect and the calcium defect of *bbp* mutant embryos, confirming that MAPKAPK2 is required in the yolk cell (n = 20, 100% rescued). Injection of 1.5 ng of mutant MAPKAPK2 mRNA (75-fold overexpression compared to WT mRNA) had no rescuing activity (n = 75). Furthermore, injection of mutant MAPKAPK2 mRNA (250 pg to 1.25 ng, n>50) into WT embryos caused no phenotypic consequences, indicating that the mutant protein is inactive and has no dominant-inhibitory activity when overexpressed. In conjunction with rescuing amounts of MAPKAPK2 mRNA, we injected a translation blocking morpholino to MAPKAPK2 into *bbp* mutant embryos. Rescue was inhibited by the morpholino (n = 17, 100% no rescue; 100% rescued by mRNA injection alone), suggesting that it can block translation. However, morpholino injection alone into WT embryos did not phenocopy *bbp* (n = 14), possibly due to maternally-supplied MAPKAPK2 protein or high maternal levels of transcript.

MAPKAPK2 is a well-characterized direct target of p38 MAP kinase (MAPK). p38 MAPK activates MAPKAPK2 by phosphorylating key residues on MAPKAPK2. MAPKAPK2 has been extensively studied in cell culture, including structure function analysis, and the identification of nuclear and cytoplasmic targets [31]. However, very few of the results from these cell culture studies have been tested in an animal model.

### Subcellular Localization of Bbp Mutant MAPKAPK2

Studies of mammalian MAPKAPK2 in cell culture show that the protein is localized to the nucleus under basal conditions, but upon phosphorylation by p38 MAPK, an overriding nuclear export signal (NES) stimulates its export to the cytoplasm [29],[30],[32],[33]. The carboxy-terminal truncation of MAPKAPK2 in *bbp* mutants results in loss of the NLS (Fig. 6A), suggesting that the mutant protein may be constitutively localized to the cytoplasm and fail to phosphorylate nuclear targets, thus causing its loss of function. To address this question, we examined the subcellular localization of myc-tagged WT and Bbp MAPKAPK2 proteins in embryos. The myc-fusion did not compromise MAPKAPK2 activity, as it fully rescued the *bbp* mutant phenotype (Fig. 6E, n = 108, 100% rescued). We found that WT myc-MAPKAPK2 predominantly localized to the nucleus, with additional weak localization in the cytoplasm, consistent with previous studies in mammalian cells (Fig. 6F, n = 12). Bbp myc-MAPKAPK2 showed increased localization to the cytoplasm and the cell cortex. Surprisingly, a significant amount of the mutant fusion protein remained in the nucleus, despite loss of the NLS (Fig. 6G, n = 10). GFP-fusions to WT and the Bbp mutant proteins behaved similarly, except that the WT GFP-fusion was found exclusively in the nucleus (data not shown). The inability of the mutant fusion proteins to rescue the mutant phenotype (myc-Bbp, n = 14; GFP-Bbp, n = 14) despite their nuclear localization, indicates that other properties of MAPKAPK2 are deficient in the Bbp protein. These results also suggest that interacting factors or additional features of MAPKAPK2 can localize it to the nucleus in the absence of the NLS.

### Bbp MAPKAPK2 Lacks Kinase Activity

We next investigated the kinase activity of the mutant protein to determine if it was constitutively active in the cytoplasm or otherwise misregulated. We analyzed the phosphorylation status of a well-established cytoplasmic substrate of MAPKAPK2, heat shock protein 27 (HSP27) [31]. Antibodies specific to the phosphorylated form of HSP27 did not detect the endogenous zebrafish protein in embryos. Therefore, we analyzed the activity of WT and Bbp MAPKAPK2 in transfected HeLa cells. We first confirmed that the subcellular localization of the proteins in HeLa cells recapitulated that seen in intact embryos (data not shown). We next examined their ability to induce phosphorylation of HSP27. As shown in Fig. 6J (top panel), the Bbp protein was expressed at levels comparable to the WT protein, suggesting that the stability of the mutant protein is not grossly affected. Expression of WT MAPKAPK2 induced robust phosphorylation of endogenous HSP27 (Fig. 6J, bottom panel). Unexpectedly, Bbp was significantly impaired in its ability to induce phosphorylation of HSP27, despite the fact that its kinase domain is intact, as well as the three predicted MAPK phosphorylation sites (Fig. 6A, J). These results indicate that the Bbp mutant protein is defective in its kinase activity, causing its loss-of-function phenotype.

To test directly if MAPKAPK2 kinase activity is required for it to regulate epiboly, we generated a myc-tagged full-length kinase dead protein by changing the critical lysine in the catalytic domain to a methionine (K73M) [34], and assayed activity in both cell culture and zebrafish embryos. As expected, kinase-dead MAPKAPK2 failed to induce phosphorylation of HSP27 in a HeLa cell culture assay (Fig. 6I, bottom panel). In *bbp* mutant embryos, injection of 200 pg of the kinase dead MAPKAPK2, although stably expressed in the embryo (Fig. 6K), failed to rescue the mutant phenotype (n = 99, Fig. 6B), in contrast to the WT protein (Fig. 6E). These results demonstrate that the kinase activity of MAPKAPK2 is required for its function in epiboly, indicating that the Bbp mutant protein fails to phosphorylate a critical target in the yolk cell that regulates epiboly.

### p38 MAPK Required for MAPKAPK2 Function

The carboxy-terminal region truncated in Bbp also contains a p38 docking site, which is important for p38 to phosphorylate MAPKAPK2 efficiently [35],[36]. The lack of kinase activity of the Bbp mutant protein may be due to failure of p38 to efficiently phosphorylate and thus activate the Bbp protein. To investigate if p38 regulates MAPKAPK2 activity, we mutated the three p38 phosphorylation sites of MAPKAPK2 [29], [30], [37]–[39]. Based on the mammalian MAPKAPK2 structure, zebrafish MAPKAPK2 is expected to be phosphorylated on Threonine 202, Serine 252, and Threonine 315 by p38 MAPK (Fig. 6A). We mutated these three residues of zebrafish MAPKAPK2 to Alanines, any two of which when mutated in cell culture cause a failure in MAPKAPK2 activation [37]. In contrast to these cell culture studies, we found that the T202A/T315A double mutant MAPKAPK2 rescued *bbp* mutant embryos (40 pg, 15/16 rescued; 90 pg, 47/47 rescued). However, injection of 150 pg of the triple phospho-mutant RNA, although stably expressed (Fig. 6H, K), failed to rescue the mutant phenotype (n = 70), indicating the importance of these three sites in MAPKAPK2 function in epiboly. We also investigated the activity of the MAPKAPK2 phosphorylation site mutants in our HeLa cell culture assay and found similar results to the *bbp* mutant rescue data (Fig. 6I).

Thus, our results show that Ser252 is sufficient in the zebrafish embryo and HeLa cells for MAPKAPK2 protein function, contrasting previous studies in other cell culture systems. Considering that the three p38 phospho-residues are conserved in all MAPKAPK2 genes, we postulate that one or more of these residues is required to activate MAPKAPK2 depending on the cellular context. Taken together, we postulate that the lack of kinase activity of the truncated Bbp MAPKAPK2 is due to a loss of the p38 docking site in the Bbp mutant protein, resulting in an inability of p38 to bind, phosphorylate, and thus activate the mutant protein.

**Figure 7:**
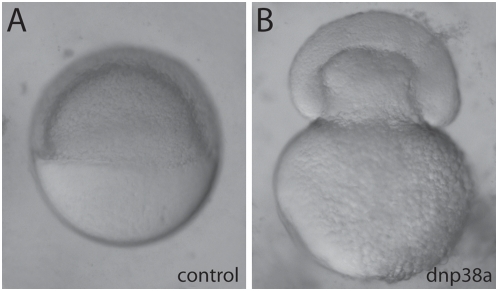
Microinjection of DNp38a mRNA phenocopies *bbp*. WT embryos microinjected with 250 pg of DsRed2 mRNA (A) or DNp38a mRNA (B), which causes a *bbp* phenocopy.

To test directly if p38 MAPK could be the upstream activator of MAPKAPK2, we investigated p38 function in epiboly progression by expressing a dominant negative p38a (DNp38) in WT embryos. *p38a* is expressed maternally and throughout blastoderm stages in the zebrafish [40],[41]. Microinjection of 250 pg of DNp38a mRNA caused 60% of the embryos to display a phenotype similar or identical to that of *bbp* mutant embryos (Fig. 7A,B, n = 155). In time-lapse microscopy analysis shimmying movements were observed in DNp38-injected embryos, similar to those seen in *bbp* mutants. Likewise, at or slightly after 50% epiboly, the blastoderm margin constricted strongly, followed by the yolk cell bursting (Video S6). These results support a role for p38 MAPK in regulating the activity of MAPKAPK2 in epiboly in the zebrafish.

## Discussion

The coordination of cell movements during developmental processes such as epiboly is not well understood. Although a fundamental cell movement process, few molecular components regulating epiboly have been described. Those that have been described are isolated components, not yet integrated into gene pathways. Here we identified a novel function for MAPKAPK2 and p38 MAPK in modulating this morphogenetic process in the early zebrafish embryo. MAPKAPK2 is required to prevent the premature constriction of the blastopore observed in *bbp* mutants. p38 MAPK is also required in this process, since mutating the three p38 MAPK phosphorylation sites in MAPKAPK2 abrogates its function and a dominant negative p38 MAPK phenocopies the *bbp* mutant phenotype. These results indicate that p38 MAPK regulates MAPKAPK2 activity during epiboly in zebrafish through the well-known MAP kinase cascade pathway.

Loss of MAPKAPK2 or p38 MAPK function causes premature constriction of the blastoderm margin, which ruptures the yolk cell and causes lethality. We can block yolk cell rupture by incubation in hypertonic media (Fig. 3 L); however, the marginal constriction persists in these conditions, indicating that the rupture and constriction are not simply due to a defect in yolk cell membrane integrity. We show that MAPKAPK2 functions within the yolk cell and postulate that it modulates actin-based contractility to close the blastopore during epiboly. The blastopore is at its greatest circumference at 50% epiboly (Fig. 1). Following that stage, the blastoderm cells migrate uniformly vegetally causing the blastopore circumference to continuously decrease in a purse-string like fashion until the blastopore is completely closed at 100% epiboly. Electron microscopy studies in *Fundulus* reveal an actin ring in the YSL adjacent to the blastoderm margin that is postulated to act as the strings during blastopore closure [15]. In zebrafish an F-actin band is first evident at 50% epiboly in a similar location in the YSL [16]. This actin band is associated with an active form of myosin, phosphorylated-myosin 2 [17], indicating the presence of an actin-myosin contractile activity in the YSL margin. This actin band is also associated with endocytosis [16], which removes the yolk membrane as the advancing blastoderm/EVL cells move vegetally over the yolk during epiboly and may be a driving force in their vegetal movement. The most intense region of this F-actin band associates with the leading edge of the EVL cells and is implicated, together with the Misshapen kinase in regulating the constriction of the marginal edge of EVL cells as they advance vegetally [17].

Pharmacological inhibitors of actin or myosin can slow the later stages of epiboly in zebrafish, implicating actin function in epiboly progression [16]. Higher doses of these inhibitors can arrest epiboly, but also cause either a dissociation of the EVL and blastoderm cells or yolk herniation due to loss of the vegetal actin mat that maintains the yolk integrity [16],[42]. We tested several actin or myosin inhibitors (cytochalasin B and D, Latrunculin A and B, blebbistatin) for their ability to suppress the marginal constriction in *bbp* mutants at 50% epiboly, however, none of them can suppress the phenotype at doses that slow epiboly in WT embryos (data not shown). Higher doses of some inhibitors can arrest epiboly in WT embryos and block the very strong constriction in *bbp* mutants; however, due to the many other roles that actin plays in development, including cytokinesis, cell adhesion, and general cell integrity, higher doses arrest development in general and cause lethality (data not shown), precluding our ability to block specifically the yolk cell constriction.

Loss of Mtx2, a predicted transcription factor, by morpholino knockdown in zebrafish results in reduction of the YSL punctate actin band and arrest in the vegetal movement of cells at the 50% epiboly point [22],[23]. Interestingly, *mtx2* morphants also constrict around the margin with a very similar phenotype to *bbp* mutants [23]. However, the temporal progression of the constriction in *mtx2* morphants coincides with the normal timing of blastopore closure in a WT embryo. That is, the margin constricts as if epiboly were progressing normally, although the cells fail to move vegetally. This contrasts the marginal constriction in *bbp* mutant embryos, which occurs rapidly during a 20 to 30-minute window, rather than over a 3-hour time period. The reduced punctate actin band in *mtx2* morphants may result in epiboly arrest. While the remaining strong F-actin at the EVL margin may mediate the marginal constriction observed in *mtx2* morphants and may be precociously activated in *bbp* mutant embryos.

In zebrafish, endogenous calcium release activity, as well as a requirement for calcium during epiboly, supports the importance of calcium signaling in epiboly progression [16]. Calcium levels are low during early epiboly [24]–[26],[43], but increase and become dynamic from 50 to 100% epiboly. Spikes of calcium are evident in the yolk cell beginning at 50% epiboly, followed by waves of calcium that traverse the blastoderm margin from 65% epiboly to blastopore closure [16],[24]. Loss of calcium causes a loss of the yolk cell actin band and a blockage in epiboly progression [16]. Considering that calcium positively regulates actin contraction [44], the dramatic increase in calcium release observed in *bbp* mutant embryos (Fig. 4) is consistent with increased actin contraction causing the abnormal morphogenesis movements. During early epiboly when calcium release is normally infrequent, we observed striking calcium dynamics in *bbp* mutants, coincident with the abnormal shimmying movements observed prior to 50% epiboly, suggesting abnormal F-actin contractile movements prior to 50% epiboly. Furthermore, we observed increased and sustained levels of calcium at the margin, when morphological constriction is apparent. The constriction phenotype is remarkable in its precise timing at specifically the 50% epiboly point in all mutant embryos, coincident with the timing of robust F-actin band formation at the YSL margin. We postulate that only when the F-actin band fully forms at 50% epiboly in conjunction with EVL marginal F-actin does the abnormal calcium regulation cause lethality through an unregulated F-actin constrictive force.

One well characterized target of MAPKAPK2, HSP27, plays a positive role in actin polymerization when phosphorylated by MAPKAPK2 [45]–[49]. Our studies suggest that actin polymerizes normally in *bbp* mutants, since the yolk cell actin band forms at 50% epiboly in *bbp* mutants. To our knowledge, there are no known MAPKAPK2 targets that inhibit actin-myosin contraction or calcium release. Thus, our results suggest a novel target of MAPKAPK2 that normally restricts sustained actin constriction to regulate tissue morphogenesis.

Although well studied in cell culture, MAPKAPK2 has been little studied in model organisms. While a MAPKAPK2 gene exists in both Drosophila and C. elegans, no mutant alleles have been reported. RNAi screens in Drosophila cell culture suggest a role for the fly homolog in cell cycle progression and cell shape regulation [50],[51].

In the mouse a targeted mutation of MAPKAPK2 is viable and fertile, but exhibits defects in mediating inflammatory responses [52]–[54]. Double mutants of MAPKAPK2 and the closely related MAPKAPK3 in the mouse display more severe defects in the inflammatory response, but do not exhibit developmental abnormalities, although they are widely expressed in development [55]. The third less related subfamily member, MAPKAPK5, exhibits an incompletely penetrant embryonic lethal phenotype in the mouse [56]. Generation of double and triple mutants of these MAPKAPKs will be required to reveal potential overlapping functions. Zebrafish have a duplicate MAPAPK2 gene that is not expressed until 3 dpf (E. Brito and DSW, unpublished observation). Zygotic roles for MAPKAPK2 in zebrafish development may be masked by the duplicate homolog or by the activity of homologs of the other family members MAPKAPK3 and/or MAPKAPK5, since MAPKAPK2/*bbp* homozygous zygotic mutants in zebrafish are viable to adulthood, with no obvious developmental defects.

The dramatic cell movements driving epiboly are crucial to the development of the body plan of anamniote vertebrates. We show that the p38 MAPK pathway is a critical component in regulating this process within the teleost yolk cell. Future studies will be required to reveal the mechanism by which this pathway regulates blastopore closure in this fundamental cell movement process. The early requirement for MAPKAPK2 and the accessibility of the zebrafish embryo will provide an excellent in vivo model for investigating the function and regulation of MAPKAPK2, which has primarily been studied in cell culture. In particular understanding the role of MAPKAPK2 during zebrafish epiboly will be valuable in the identification of MAPKAPK2 inhibitors to modulate the inflammatory response that MAPKAPK2 mediates in chronic inflammatory conditions in humans.

## Materials and Methods

### Ethics Statement

The animal work in this study was approved by the Institutional Review Board of the University of Pennsylvania School of Medicine.

### Phenotypic Characterization

All analyses were performed with the *bbp^p58cd^* allele [18]. Optimized fixation protocols for the cytoskeletal proteins were followed. Tubulin was visualized using KMX-1 (Boerhinger Mannheim) and F-actin with rhodamine-phalloidin (Molecular Probes) as described [57]. Myc-tagged protein was visualized by fixing embryos in 4% PFA in PBS, blocking with 5% BSA and 0.5% Tween-20 in PBS and staining with anti-myc monoclonal antibody 9E10 (University of Pennsylvania, Cell Center Service Facility), followed by confocal microscopy. *In situ* hybridization was performed using *gsc* and *no tail* [58], *eve1* [59], *bmp4* [60], and *foxb1.2* (formerly *fkd3*) [20].

Endocytosis was analyzed by incubating manually dechorionated embryos in 1% rhodamine-dextran (MW 10,000, Molecular Probes) in E3 medium from sphere stage to 50% epiboly as described [8],[61]. Fixed embryos were analyzed by confocal microscopy.

For yolk cell membrane integrity studies, WT and *bbp* embryos were incubated in hypertonic Ringers solution (116 mM NaCl, 2.9 mM KCl, 0.8 mM MgSO_4_, 1.8 mM CaCl_2_, 5 mM HEPES) from the 4-cell stage through 24 hpf.

### Blastoderm Transplants

Whole blastoderm transplants were performed as previously described [27]. Briefly, mutant and WT embryos were injected at the one-cell stage with 1 nl 2.5% rhodamine-dextran (MW 10,000, Molecular probes) and 1 nl 0.05 mM Sytox Green (Molecular Probes), respectively, in 0.1 M KCl. Embryos were dechorionated and kept in E3 medium until the 1000-cell stage. Transplants were performed in 1× Ringers solution (116 mM NaCl, 2.8 mM Kcl, 5 mM HEPES, 1 mM CaCl_2_, pH 7.2) containing 1.6% whipped and cleared chicken egg whites. Blastoderms were separated from yolks using a glass knife and hybrid embryos formed by placing blastoderms onto naked yolks using slight pressure until adhered, then transferred to 1/3× Ringers solution after 15 minutes and analyzed.

### Mapping, Molecular Identification and Cloning of *bbp*

Genomic DNA was pooled from mutant and WT sibling females, and the *bbp* mutation, *p58cd* [18], mapped to a chromosomal position using SSLP markers spaced throughout the genome, as described [19],[28]. SSLP markers flanking the mutation (z22766 and z22355) were used to genotype individual fish. Fish were generated for fine mapping by crossing *bbp*−/+ females to *bbp*−/− males. Both *bbp*−/+ and *bbp*−/− females were scored for recombination between markers z22766 and z22355.

cDNA was made from ovary RNA purified from *bbp*−/− and *bbp*+/+ fish using Superscript II (Invitrogen). MAPKAPK2 (MK2) cDNA was amplified using the primers: 5′-CCATCGATGGGTGTTGCCAAAGAAAGAC-3′ and 5′-GCTCTAGATCCACCGAGTTATTGCTTCC-3′. The product was sequenced and cloned into Cla1 and Xba1 sites in pCS2+. Full-length WT and *bbp* MK2 were cloned into a pCS2+ vector containing six tandem copies of a myc epitope (gift of Dr. Peter Klein), yielding an N-terminal myc-fusion protein. Kinase-dead myc-MK2 (K73M) was made by site-directed mutagenesis using the primers: 5′-GTGGGGAGAAGCTCGCTTTAATGATGCTTCATGACTGCCCAAA-3′ and 5′-TTTGGGCAGTCATGAAGCATCATTAAAGCGAGCTTCTCCCCAC-3′. Non-phosphorylatable myc-MK2 (T202A, S252A, T315A) was made by site-directed mutagenesis using the following primers in three subsequent reactions: 5′-ACACACAACTCTCTGGCCGCCCCCTGCTATACTCCTTATTAT-3′ with 5′-ATAATAAGGAGTATAGCAGGGGGCGGCCAGAGAGTTGTGTGT-3′ (T202A), 5′-GAATCATGGATTGGCAATTGCTCCTGGTATGAAGAAACGAAT-3′ with 5′-ATTCGTTTCTTCATACCAGGAGCAATTGCCAATCCATGATTC-3′ (S252A), and 5′-CAATCAATGGAGGTTCCACAGGCACCCCTACACACCAGCCGT-3′ with 5′-ACGGCTGGTGTGTAGGGGTGCCTGTGGAACCTCCATTGATTG-3′ (T315A). DNp38a (T181A, Y183F) was generated from a WT p38a cDNA in pCS2+ (gift of T. Hirano) by site directed mutagenesis with the primers 5′-GACACACAGATGATGAGATGGCCGGCTTTGTGGCCACAAGGTGGTATC-3′ 5′-GATACCACCTTGTGGCCACAAAGCCGGCCATCTCATCATCTGTGTGTC-3′. Capped mRNA was produced from pCS2+ constructs with *m*Message *m*Machine (Ambion) and injected into embryos, as previously described [62]. A MK2 morpholino, GTTGGCGTTAGTCAACATCTCCCAC (Gene Tools, Philomath, Oregon) was injected at 5 ng/nl in 0.1 M KCl at the one-cell stage.

For *in situ* hybridization of MK2, a probe was generated from the pCS2+MK2 vector digested with HinDIII and transcribed with T7 polymerase. SacII digestion, followed by SP6 polymerase synthesis generated the sense control probe.

### Cell Culture and Western Blotting

HeLa cells were grown in Dulbecco's modified Eagle's medium (DMEM) supplemented with 10% fetal bovine serum (FBS), penicillin and streptomycin, and GlutaMax (Gibco BRL) to a density of 2×10^5^ per 35 mm plate at 37°C in 5% CO_2_. Cells were transfected with tagged and untagged MK2 WT and mutant plasmids, as described [63]. Cell lysates were analyzed by a standard Western blot protocol using anti-MK2, anti-HSP27, anti-Phospho-HSP27 (Assay Designs), and anti-Phospho-MK2 (Cell Signaling). Zebrafish embryonic proteins were resolved as described [64] with each lane containing 2 embryo equivalents. Western blotting was performed with anti-myc monoclonal antibodies.

### Imaging, Time-Lapse Microscopy and Confocal Analysis

Still images of live embryos and *in situ* hybridization were captured with iVision (DVL Software). For time lapse-microscopy, embryos were immobilized in individual chambers in 1% agarose in E3 and covered by 3% methylcellulose in E3. Time-lapse movies were created using OpenLab (Improvision, Beverly, MA). Confocal analysis of tubulin and phalloidin staining, endocytosis, and MAPKAPK2 localization was performed using a Zeiss confocal and LSM510 software. For *in vivo* calcium imaging, 1-cell stage embryos were microinjected with Fura-2 Dextran or Bis-Fura2 (Molecular Probes) and imaged as described [65]. Image pairs were collected at 15-second intervals through epiboly stages. For rescue of the calcium defect, MAPKAPK2 RNA (90 ng/ul) co-mixed with Texas Red (TxR) or TxR alone as a control was injected in the yolk cell below the blastoderm at the 512-cell stage. Periodically frames were collected at 560 nm to locate the TxR lineage marker and *in vivo* calcium imaging performed as above. For YSN labeling, embryos were injected in the YSL at 1000-cell stage with 1 nL of 0.25 mM Sytox Green in 0.1 M KCl. Embryos were mounted in 0.12% low melt agarose. Images were acquired on a Zeiss Axiovert 200 and processed in Axiovision software. For time-lapse movies, images were processed in Photoshop, ImageJ and Quicktime Pro.

## Supporting Information

Video S1 Shimmying movements in the embryo occur prior to the constriction and bursting of the yolk. Time-lapse movie of WT (left panel) and *bbp* (right panel) embryo beginning at high stage. The *bbp* embryo exhibits embryo-wide shimmying movements that gain in amplitude prior to the constriction at 50% epiboly.
(7.30 MB MOV)

Video S2 *bbp* YSN display normal movements until marginal constriction. Animal view of WT (left panel) and age-matched mutant (right panel). I-YSN rapidly contract to a small area of the I-YSL.
(1.03 MB MOV)

Video S3 *bbp* YSN display normal movements until marginal constriction. Lateral view of WT (left panel) and age-matched mutant (right panel). I-YSN rapidly contract to a small area of the I-YSL.
(0.70 MB MOV)

Video S4 WT embryos display controlled and consistent calcium release during epiboly. Time-lapse movie of WT embryo from early epiboly through 50% epiboly. Calcium release is visualized by fura-2 and indicates the intensity of the release (stable levels of calcium, cool colors indicating low intensity; dynamic levels of calcium, warm colors indicating high intensity). WT embryos maintain overall stable calcium release levels throughout early epiboly.
(5.50 MB MOV)

Video S5 *bbp* embryos display increasingly dynamic calcium release prior to the bursting of the yolk. Time-lapse movie of *bbp* embryo from early epiboly through bursting at 50% epiboly. Calcium release is visualized by fura-2 and indicates the intensity of the release, as in Movie 4. Ectopic calcium release increases dramatically over time as seen by sparks of high intensity. Frequency and intensity of the sparks increased simultaneously with the intensity of the shimmying movements in the embryo and increased release at the cell margin, culminating in the lethal bursting of the yolk.
(7.07 MB MOV)

Video S6 DNp38a mRNA injection causes bbp phenocopy. (Top 3 rows) 100 pg DNp38a mRNA injected embryos, (bottom row) buffer control injected embryos. Nine of 12 DNp38 injected embryos display yolk cell rupture phenotype. 5 minute intervals.
(9.31 MB MOV)
